# Mixture toxicity assisting the design of eco-friendlier plant protection products: a case-study using a commercial herbicide combining nicosulfuron and terbuthylazine

**DOI:** 10.1038/s41598-018-23883-5

**Published:** 2018-04-03

**Authors:** Libânia Queirós, Tânia Vidal, António J. A. Nogueira, Fernando J. M. Gonçalves, Joana Luísa Pereira

**Affiliations:** Department of Biology, CESAM (Centre for Environmental and Marine Studies), Campus de Santiago, 3810-193 Aveiro, Portugal

## Abstract

The development of environmentally friendly plant protection products (PPPs), including pesticides, is a challenge nowadays. A commercial herbicide combining terbuthylazine and nicosulfuron as active substances (a.s.) was selected as a model PPP. The suitability of manipulating the ratio between a.s. towards alternative formulations with reduced impacts in a non-target indicator (*Lemna minor*) was assessed. The efficacy of such eco-friendlier a.s. ratios was then assessed using a target weed, *Portulaca oleracea*. Single and mixture toxicity testing with *L. minor* revealed an antagonistic joint action of the a.s., suggesting an environmentally protective effect of the combination compared to single dosing of a.s. The efficacy testing against the target weed of single and combined treatments of the a.s. showed that (i) the a.s. behave antagonistically throughout the whole *P. oleracea* response surface; (ii) there were no environmentally safe a.s. combinations ensuring target-efficacy; (iii) terbuthylazine alone was effective in controlling *P. oleracea* with no environmental hazardous potential, dosed at concentrations 10-fold lower than those involved in commercially recommended application doses. Overall, this case-study suggests that modelling tools widely used in the field of environmental risk assessment of PPPs may also have application in PPP design stages for a more efficient meeting of efficacy and environmental friendliness requirements.

## Introduction

Agriculture relies on plant protection products (PPPs) to ensure improvements in quality and yield of crops^1,2^. However, the use of PPPs may involve risks to human and environment health, reflecting into significant costs^3,4^. Regulatory agencies worldwide have already recognized this problem and have been developing tight screening protocols before the marketing of PPPs can be authorized, as well as supporting tools. Amongst these latter, modelling tools have been developed addressing PPP transport and inputs in aquatic systems. In the European Union, for example, the FOCUS platform is used to assess Predicted Environmental Concentrations (PEC values) in surface and groundwater, depending on PPP application doses and their physicochemical properties. PEC values can be compared with ecotoxicological benchmarks retrieved following ecotoxicity tests, allowing conclusions on PPP environmental hazard potential^5,6^. Still, PPPs have been reaching surface water through different transport pathways, e.g. runoff and leaching, often causing hazardous contamination scenarios^7–10^, with exposure to pesticide residues being often clearly linked to significant ecosystem risks^7,8,11–13^.

In order to counteract these environmentally hazardous scenarios, the agrochemicals industry has been impelled to innovate in the formulation of its products^14^. Common strategies to develop eco-friendlier PPP formulations include (i) the use of natural products or greener equivalents in PPP formulations^15,16^; (ii) the improvement of PPP application techniques and target delivery^17–20^; (iii) and the combination of already licensed active substances (a.s.)^21^, based on a putative synergic behavior that intends the use of lower a.s. quantities with the same levels of efficacy against the target. This latter solution is often used also to improve the control of a broader range of weeds^22^ but the success of such an approach is controversial. Whether some argued that synergic behavior can be reached, experimental evidence exists that pesticide mixtures rarely result in synergic effects^23^. Additionally, the interactive effects between formulants should be considered while assessing the toxic potential of each formulated PPP, rather than focusing only each single formulant,to predict joint action^24–27^. This is feasibly achievable using well-developed and established mixture toxicity assessment tools currently available (e.g.^28,29^). This rationale motivated the present study, where we focused on the manipulation of the ratio between two a.s. of a commercial herbicide to preliminarily evaluate the suitability of this strategy as an additional approach to formulate eco-friendlier products.

This commercial herbicide is the 2-way formulation Winner Top® (Selectis®, Portugal), with nicosulfuron and terbuthylazine as a.s. plus undisclosed formulants^20,30^, applied in crops to control weeds such as *Portulaca oleracea* and *Amaranthus* spp. Its action is systemic and residual following a single application per year, the effects in target weeds being perceptible 7–10 days after the application through a visible weeds control^31^. Nicosulfuron and terbuthylazine belong to sulfonylureas and 1,3,5-triazines chemical groups, respectively. Nicosulfuron prevents the growth of susceptible plants by blocking the synthesis of branched-chain amino acid through the inhibition of acetohydroxyacid synthase, and terbuthylazine inhibits photosynthesis by acting as a photosystem II blocker^32^.

*Portulaca oleracea*, commonly known as purslane, was selected as a representative target weed within the study since this species is a major target of the focused commercial herbicide as defined in the product documentation^31^. This is a major worldwide weed affecting several crops, including maize, rice, wheat, cotton and sugarcane^33^, which contributes to its representativeness as a test species. Adding to its small size, rapid growth and tolerance to a relatively wide range of culturing conditions^33^, purslane is easy to find in seeds given that there is some tendency to cultivate the species for its medicinal properties and traditional use as salad and soup vegetable^34^, all contributing for the species suitability for handling and testing in the laboratory. Taking into account the susceptibility of freshwater ecosystems to PPP residues contamination (see above), the macrophyte *Lemna minor* was selected as the non-target test species to address the possibility of establishing eco-friendlier alternatives to the commercial a.s. combination. This option was ruled by (i) the established status of *Lemna sp*. as standard ecotoxicological test species^35^; (ii) the herbicidal and systemic nature of the PPP, which *a priori* suggests that macrophytes should be more sensitive (thus more environmentally protective) than non-plant indicators and equivalent indicators lacking a vascular system such as microalgae; (iii) the available database on the ecotoxicity of each a.s. confirming *Lemna* sp. as a very sensitive species^32,36,37^.

Given that only the a.s. terbuthylazine and nicosulfuron are disclosed as active components of the model commercial formulation, these were the components considered for testing within the present study, which was structured following a tiered approach through the accomplishment of sequential specific aims. In a first tier, the response of the non-target *L. minor* was assessed following single exposure to each a.s. to feed reference mixtures toxicity models of Concentration Addition (CA^38^) and Independent Action (IA^39^), and further prediction of mixture toxicity response surfaces. CA assumes that mixture components act as dilutions of each other since they have a similar toxicological mode of action, while IA assumes that the components of a mixture act independently through a dissimilar mode of action, thus the effect of one of the components in the mixture should remain unchanged in the presence of another component (see details on the mixture toxicity theory in e.g.^28,29,40^). Responses of *L. minor* to mixtures as predicted by these reference models allowed the definition of the mixture treatments for further testing, triggering the second tier of the study. Here we aimed specifically at defining the actual response surface of *L. minor* following exposure to the mixture of terbuthylazine and nicosulfuron towards spotting deviations (synergism/antagonism, dose-level and dose-ratio^29^) from the reference models of mixture toxicity. The least hazardous mixtures between the a.s. (e.g. antagonistic combinations) would represent eco-friendlier alternatives to the ratio between a.s. used in the commercial formulation. The third tier of the study then aimed at testing the efficacy, towards the target weed *P. oleracea*, of these eco-friendlier mixtures of nicosulfuron and terbuthylazine, thus validating their suitability to control a model weed.

## Results and Discussion

**Figure 1:**
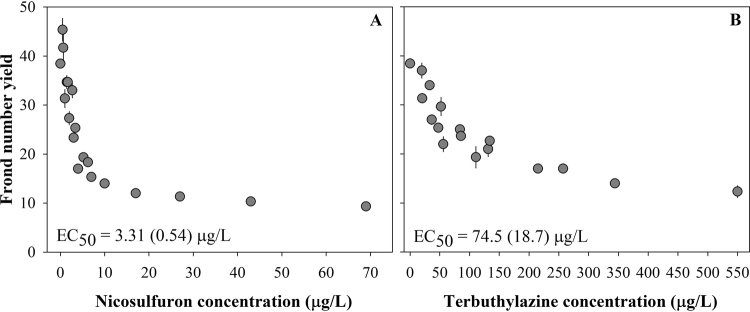
Effects of nicosulfuron (**A**) and terbuthylazine (**B**) dosed singly in mean *Lemna minor* frond number yield, with error bars representing the standard error (n = 3). The allosteric decay model was fit to the raw data to feed mixture toxicity models and to calculate EC_*x*_ values; EC_50_ values and corresponding 95% confidence interval within brackets is shown for indicative purposes, while EC_1_, EC_5_ and EC_20_ can be found in Table S1 as they were used as references for establishing mixture treatments tested with *Portulaca oleracea*.

The response of *L. minor* to single exposures allowed to define the mixture scheme for the second tier of the study, and provided an overview on the relative toxicity of the mixture components. Single-chemical concentration-response curves were well covered by experimental data (Fig. 1) and significant fitting of the nonlinear decay model to the experimental results was achieved (F_4, 127_ = 237.4; *p* < 0.001). Maximal inhibitory effects of 76% and 56% were reached at 69 and 257 µg/L nicosulfuron and terbuthylazine, respectively, and the higher toxicity of nicosulfuron to *L. minor* was confirmed by an estimated 7 d-EC_50_ value for frond yield inhibition (3.31 µg/L) lower by one order of magnitude compared to the corresponding benchmark of 74.5 µg/L found for terbuthylazine (Fig. 1).

This difference in toxicity - as read from an integrative endpoint such as biomass yield - between substances designed to have an efficient herbicidal action should result from the interplay between intake suitability, the metabolic pathway affected and the meaning of the impairment for the overall physiological performance of the organism. Terbuthylazine has a higher Kow than nicosulfuron (3.4 vs. 0.61 at 20 °C and pH = 7^32^), meaning that the former is much more lipid-soluble thus of easier internalization through cell membranes^41^. However, *L. minor* is a rooted macrophyte, and the translocation of substances absorbed through the roots should compensate for a lower direct intake of nicosulfuron via inner frond surface contact compared to terbuthylazine (see Cedergreen and Madsen^42^ for a revision on chemical’s absorption in *Lemna*). Thus, it is conceivable that the impairment of aminoacid synthesis by nicosulfuron can more prominently constrain *L. minor* growth than photosystem II inhibition by terbuthylazine at equivalent doses, hence the lower sensitivity of *L. minor* biomass to the latter.

**Figure 2:**
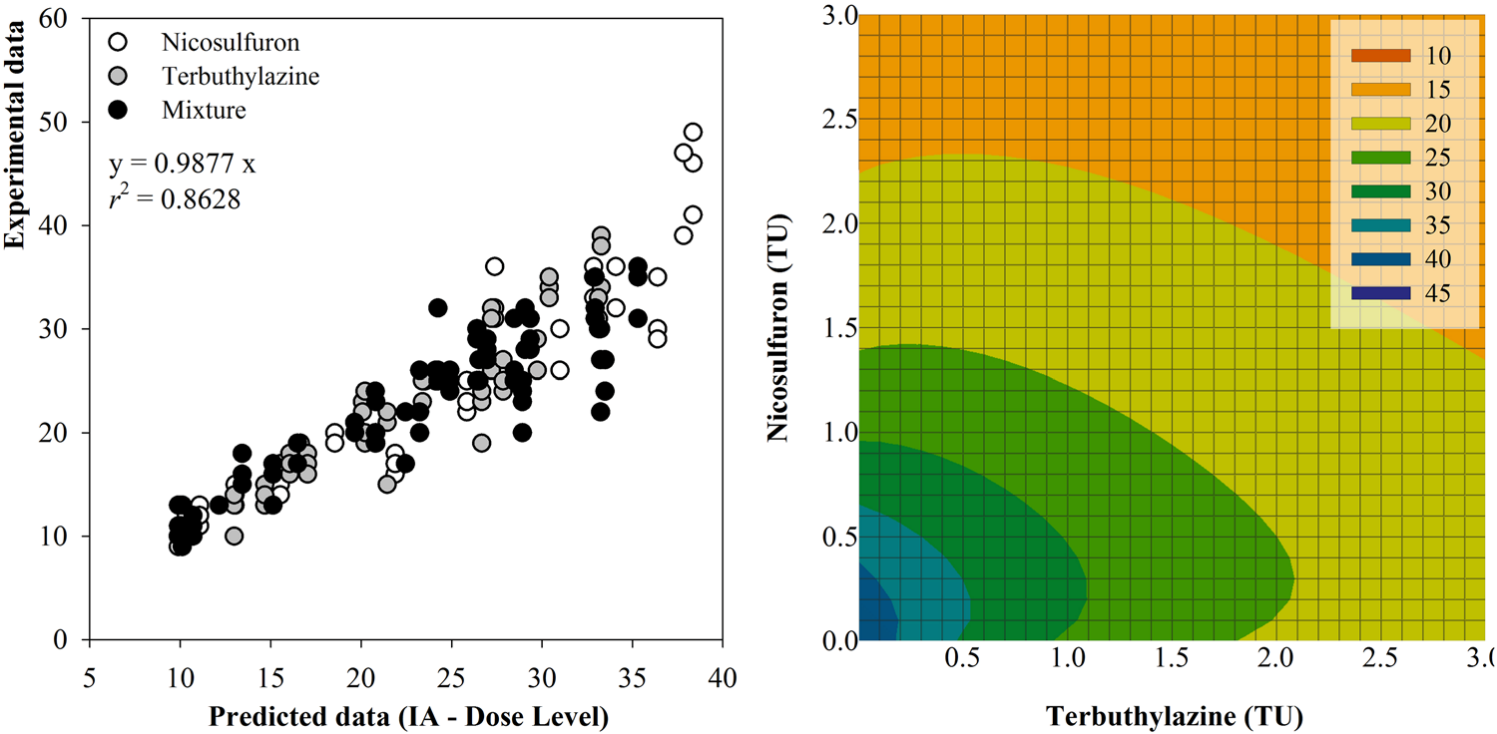
*Lemna minor* response (frond number) following a 7-d exposure to binary mixtures between nicosulfuron and terbuthylazine, according to the dose-level dependent IA model. The left-hand panel shows the agreement between experimental responses and those predicted by the model; the regression equation and corresponding coefficient are given embedded within the graph. The right-hand panel shows the isobologram illustrating the mixtures response surface as predicted by the model under a TU mixture strength scaling; the colour gradient is indicative of the level of effect, the graphical legend referring to frond number.

Taking into account different statistical parameters, the mixture toxicity model that best fitted the mixture exposure of *L. minor* to nicosulfuron and terbuthylazine was the IA model incorporating dose-level dependent effects (DL-IA; Table 1). The predictive ability of DL- IA in the present case is additionally illustrated in Fig. 2 by the strong association between observed data and their counterparts as predicted by the model. The value found for the ‘a’ parameter considering the dose-level deviation of IA was slightly above zero (0.002; see Table 1), which indicates a tendency for an antagonistic behaviour of the mixture at low dose level and a synergic behaviour at high dose level^29^. This latter was unperceived in the corresponding isobologram (Fig. 2), where antagonism throughout the whole response surface at the mixture strength range focused is rather evident by the convex shape of the isoboles. The value found for the ‘b’ parameter was heavily negative (−1231; Table 1), confirming that the shift from antagonism into synergism as expected by the DL-IA model is not likely to occur and that the magnitude of antagonism is effect level dependent^29^. The isobologram denotes that stronger antagonism (higher degree of convexity) occurred consistently at lower nicosulfuron doses (Fig. 2), which is consistent at all but the lowest effect level (bluish areas of the isobologram).

**Table 1:** Statistical parameters for the fitting of experimental data retrieved while testing mixtures of nicosulfuron (nic) and terbuthylazine (terb) to CA and IA, as well as deviations from these reference models, denoting dose-level dependence (DL), synergism/antagonism (S/A) and dose-ratio (DR) dependence. Data relative to mixture testing with the non-target macrophyte Lemna minor and the target weed Portulaca oleracea are represented.

			*r* ^2^	RMSD	SSE (df)	AIC	*P* (F-test)	a	bDL	b_nic_	b_terb_
***Lemna minor***	CA	Baseline	0.633	—	1300 (71)	230.8	<0.001	—	—	—	—
		DL	0.748	3.450	892.5 (69)	206.7	<0.001	1.100	−0.338	—	—
		S/A	0.730	3.544	954.8 (70)	209.4	<0.001	1.750	—	—	—
		DR	0.744	3.502	907.6 (68)	210.6	<0.001	1.194	—	2.869	−1.675
	**IA**	Baseline	0.595	—	1435 (71)	238.4	<0.001	—	—	—	—
		**DL**	**0.742**	**3.493**	**915.2** (**69)**	**208.7**	<**0.001**	**0.002**	**−1231**	—	—
		S/A	0.669	3.926	1171 (70)	225.2	<0.001	1.000	—	—	—
		DR	0.695	3.823	1082 (68)	224.1	<0.001	0.701	—	2.357	−1.656
***Portulaca oleracea***	CA	Baseline	0.734	—	0.548 (9)	−29.24	0.018	—	—	—	—
		DL	0.912	0.118	0.180 (7)	−32.31	0.003	20.50	−0.083	—	—
		S/A	0.912	0.114	0.180 (8)	−39.86	<0.001	22.81	—	—	—
		DR	0.912	0.123	0.180 (6)	−22.59	0.011	12.36	—	1.043	11.32
	**IA**	Baseline	0.806	—	0.400 (9)	−33.97	0.005	—	—	—	—
		DL	0.912	0.118	0.180 (7)	−32.31	0.003	7.870	−0.104	—	—
		S/A	0.912	0.114	0.180 (8)	−39.86	0.001	8.576	—	—	—
		**DR**	**0.912**	**0.123**	**0.180** (**6)**	**−22.59**	**0.011**	**4.647**	—	**0.392**	**4.255**

RMSD (Root Mean-Square Deviation) provides a measure of the difference between predicted values and those actually observed (the lower the better); SSE (Error Sum of Squares) is the sum of the squared differences between each observation and its group’s mean (the lower the better), and df stands for the residual degrees of freedom. The model best fitting the dataset in each case was highlighted bold.

Nicosulfuron and terbuthylazine are herbicides with dissimilar modes of action^32^. Because both metabolic pathways are common between target weeds and *L. minor*, and provided that this non-target species was proven sensitive to both compounds (see the above regarding single-chemical toxicity), a good description of the mixture toxicity by an IA model was somewhat expected taking into account the basics on mixtures toxicity theory (e.g.^40^). Also, although no previous studies were found regarding the toxicity of mixtures between sulfonylureas (such as nicosulfuron) and triazines (such as terbuthylazine) for a more direct discussion, antagonistic effects of pesticide mixtures are not rare. For example, they were already found following exposure of fish to glyphosate and cypermethrin^43^, of midges to pyrethroids and neonicotinoids^44^, and of bacteria to quaternary mixtures between ionic liquids structurally much resembling new-generation pesticide molecules with long carbon-based alkyl chains^45^. Despite these examples and the fact that pesticide mixtures are rarely synergic against biological targets^23^, the finding of an antagonistic behavior in the present study was somewhat surprising since we worked with the components of a PPP formulation (Winner Top®) and such formulations are, in principle, designed towards the best efficacy against the target. Under this ruling principle, it seems logical that antagonistic interaction between a.s. should be avoided. Given that *L. minor* shares with the target weed the metabolic pathways involved in their toxicity, antagonistic effects of the a.s. combination within the commercial formulation in *P. oleracea* should reasonably be expected, unless there is a susceptibility modulation by a non-identified mechanism. Although nothing could be found in the literature regarding *L. minor* or *P. oleracea*, some maize hybrid crops are tolerant to nicosulfuron^46^, which is related to different rates of absorption/translocation and/or better detoxification metabolism^47,48^.

**Figure 3:**
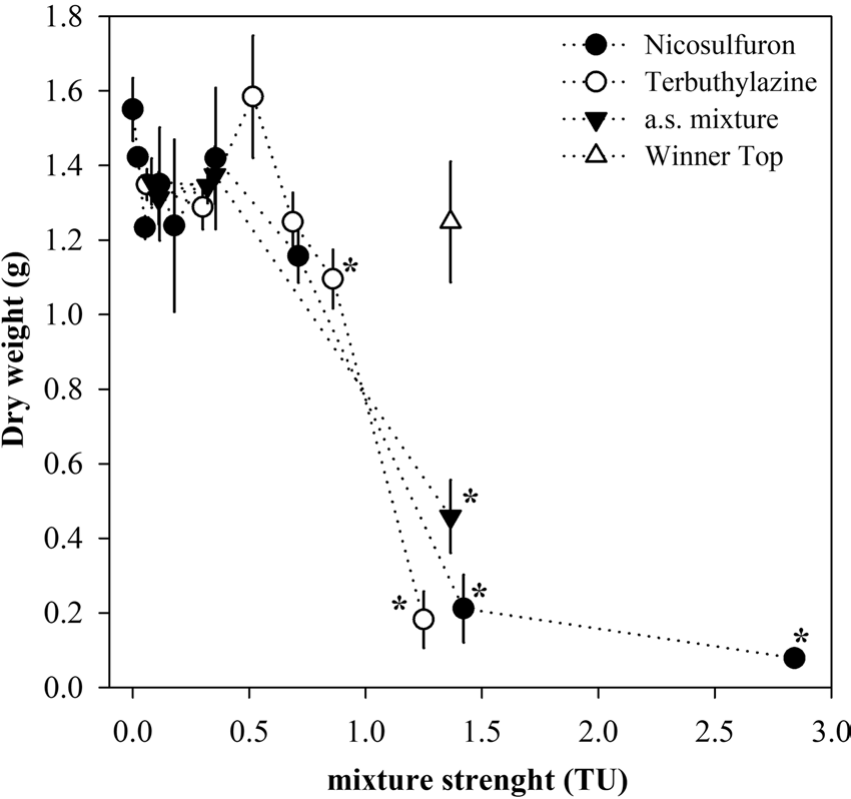
Dry weight average of *Portulaca oleracea* 16 days after treatment with nicosulfuron, terbuthylazine their binary mixtures (a.s. mixture). The effects of Winner Top® are also represented in a treatment equivalent to one of the a.s. mixtures tested. Error bars represent the standard error. The dashed lines were added for clarity purposes only. The asterisks assign significant differences in dry weight relative to the control treatment (Dunnet test; p < 0.05).

The mixture design applied to *P. oleracea* involved additional single-chemical treatments, thus allowing direct interpretation of the weed sensitivity to each mixture component (see Fig. 3 for an overview). Nicosulfuron and terbuthylazine applied singly were able to significantly depress *P. oleracea* growth (*p* < 0.001) both on the basis of dry weight (One-way ANOVA: F_10, 17_ = 15.01 and F_6, 15_ = 28.74 for each chemical, respectively; Fig. 3) and leaf number (One-way ANOVA: F_10, 17_ = 11.48 and F_6, 15_ = 8.18 for each chemical, respectively). Given that there were some difficulties while recording the number of leafs at the end of the tests, and provided that the allosteric decay model used to estimate concentration-response curves fitted better to the experimental dry weight data from single-chemical testing (*r*^2^ = 0.869; F_4, 41_ = 67.71 with *p* < 0.001), further interpretation will be based on dry weight records only. As applied singly, nicosulfuron induced significant decrease in *P. oleracea* dry weight only at 1.42 and 2.84 Toxic Units (TU) (Fig. 3), corresponding to application doses of 60 and 120 g/ha, respectively (Table S1), which translate into surface water concentrations higher than the *L. minor* EC_20_ (safety) benchmark and is inconsistent with regulatory environmental safety demands^5,6^. Terbuthylazine significantly impaired *P. oleracea* dry weight at application doses corresponding to values lower than EC_20_ to *Lemna minor* (0.85 and 1.2 TU, i.e. 50 and 72.74 g/ha; Table S1 and Fig. 3). A slightly higher sensitivity to the latter was found based on a lower 16 d-EC_50_ value estimated with non-overlapping 95% confidence intervals of 42.22 (7.87) compared to 58.22 (5.77) g/ha. This was in line to the relative sensitivity order found for the non-target indicator *L. minor*, further supporting the possibility of the occurrence of similar tendencies between species in the response to the mixture challenge.

**Figure 4:**
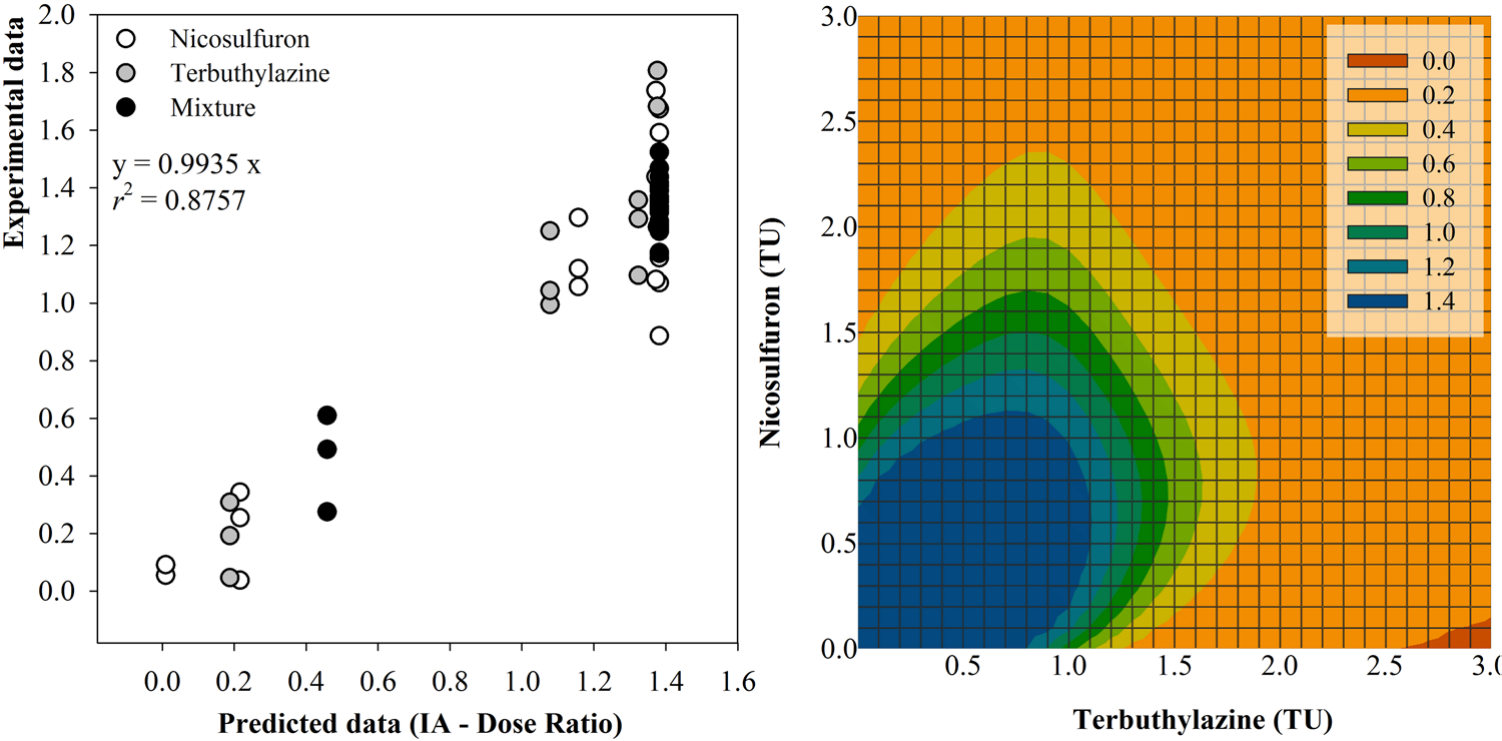
*Portulaca oleracea* response (aerial biomass, i.e. dry weight of leaves and shoots) following a 16-d growth period following treatment with binary mixtures between nicosulfuron and terbuthylazine, according to the dose-ratio dependent IA model. The left-hand panel shows the agreement between experimental responses and those predicted by the model; the regression equation and corresponding coefficient are given embedded within the graph. The right-hand panel shows the isobologram illustrating the mixtures response surface as predicted by the model under a TU mixture strength scaling; the colour gradient is indicative of the level of effect, the graphical legend referring to aerial biomass at the end of the assay.

The mixture between nicosulfuron and terbuthylazine was also able to significantly suppress the species growth (One-way ANOVA: F_10, 8_ = 13.65 with *P* = 0.001; Fig. 3). The LOEC found for terbuthylazine was the lowest compared to the counterparts retrieved for nicosulfuron and the mixture. And, at treatment strengths between 1.0 and 1.5 TU, the efficacy of terbuthylazine dosed singly (88% dry weight inhibition at 1.25 TU compared to the control) was higher than that of nicosulfuron (86% inhibition at 1.42 TU) and the equitoxic mixture combining the doses equivalent to *L. minor* EC_20_ values (70% inhibition at 1.37 TU), which was the only mixture tested that was able to significantly impair *P. oleracea* biomass yield. This suggests that there is no advantage of dosing a mixture rather than one of the a.s. singly when the intent is the control of the weed. The apparent decrease of the mixture efficacy compared to terbuthylazine dosed singly was confirmed throughout the whole response surface. The mixtures toxicity model best fitting the experimental data based on several indicators was the dose-ratio dependent IA (Table 1), and the antagonistic behaviour of the mixture throughout the modelled response surface is evidenced by the strongly convex shape of the isoboles in Fig. 4. The positive value of the parameter ‘a’ in dose-ratio dependent IA confirms that the mixture behaves antagonistically throughout the whole predicted response surface and the values found for the ‘b’ parameters denote that the antagonism is mostly due to terbuthylazine, which scored higher (Table 1^29^). Although the significant fitting of the mixture toxicity models (Table 1) was not constrained, a note is due on the establishment of the mixture treatments in this case, which lead to an uneven distribution of experimental data across the response surface preventing a proper support to the predictions in certain areas (see in the left-hand panel of Fig. 4). Still, our results confirm that the a.s. are not synergists as argued in the label of the commercial formulation Winner Top®^31^.

At this point it is reasonable to assume that a terbuthylazine alone would exert better and environmentally safer herbicidal toxicity than a two-way formulation with nicosulfuron as used in the model commercial compound Winner Top®. Terbuthylazine alone, applied at much lower concentration (at 72 g/L), would be more effective in reducing *P. oleracea* dry weight (practically to 0 g) than the concentration involved in the recommended application dose of Winner Top® (625 g/ha terbuthylazine, considering the lower application rate 2.5 L/ha), which is inherently environmentally unsafe considering that 72 g/ha delivers the *L. minor* EC_20_ in surface waters. Such overdosing recommendation in the commercial product is likely linked to the recognition that significant off-target losses may occur during application even with the great efforts to improve formulations in this sense, but this paradoxically implies a raised hazardous potential towards non-target species and edge-of-field ecosystems receiving the residues that do not reach the target^4,49–51^. On the other hand, nicosulfuron alone exerted significant toxicity towards *P. oleracea* only at 60 g/ha (Fig. 3), corresponding to a contaminant concentration in surface water (19.66 µg/L; Table S1) which is more than one order of magnitude above the *L. minor* EC_20_ safety benchmark (1.59 µg/L translating into an application dose of 4.86 g/ha; Table S1). Based on our results, a formulation composed only by nicosulfuron could be effective against the target species but disregarding environmental safety, thus it would not constitute a suitable eco-friendly alternative to the combination between the a.s. used in Winner Top®. Curiously, a formulation containing only nicosulfuron is currently marketed by Selectis® (Winner®^52^) with recommended application rates corresponding to 40–60 g/ha. Based on our results (limited by the laboratory scale used and the dosage of a.s. rather than whole formulations), no relevant effects in *P. oleracea* should be expected at these doses and they can represent significant environmental hazard.

A relevant argument counteracting the above interpretation could be given on the putative role of the formulants other than the a.s. present in the commercial formulation in reversing the antagonistic effects between the a.s. However, we tested on this and remarkably observed a much lower inhibition caused by Winner Top® compared to the equivalent mixture combination of its a.s, both combining 72.74 g/ha of terbuthylazine and 4.86 g/ha of nicosulfuron (Fig. 3, the open triangle representing the dosing of the commercial formulation while the black triangles represent the a.s. combination cleared of other formulants). Thus, the formulants other than the a.s. appear to greatly decrease the efficacy of the pesticides towards the target weed, reinforcing that the option for a mixture formulation as used in Winner Top® was not appropriate, thus possibly not accurately assessed during PPP design stages. One can only speculate on the reasoning behind the use of this formulation in the marketed product because there is no disclosed information in this arena that we could reach. PPP formulations are designed in such a way that rapid and uniform dispersion through large treatment areas with minimum amounts of active ingredient is ensured^53^. Besides these application and effectiveness features, improved handling, storage and safety (of the operator and the environment) is also taken into account^54,55^. Under this context, emulsifying concentrates were for long a primary option because they constitute chemical solutions and the formulation tends *a priori* to be stable. However, most emulsifying concentrates contain volatile aromatic solvents which are intrinsically hazardous^53,56,57^, and their replacement with new formulation types including suspension concentrates has been favoured^56^. Suspensions generally perform worst *per se* because the larger suspension particles are less likely to penetrate cuticles, and adjuvant dispersants and/or surfactants are commonly added to the formulations since they can facilitate this part of the job^56,58–60^ although the stabilization of such a system is often a challenge^56,61^.

Oil dispersions such as Winner Top® are essentially suspensions with built-in oil adjuvants. Since they join surfactants and oils as adjuvants within the formulation, better performance in the field is expected often with the advantage of dispensing tank mixing^55,62^. However, considering our results, the use of an oil dispersion formulation in Winner Top® was apparently not advantageous since the addition of adjuvants seems to lower the a.s. efficacy, impelling for the raise of their application concentrations into environmentally unsafe levels. And, as much terbuthylazine was added to the formulation (recommended application doses represent 625 g/ha terbuthylazine, which translate into 10.7 TU that score almost one order of magnitude higher than the 1.25 TU in Winner Top® treatment used in this study; Table S1 and Fig. 3), the higher the magnitude of the antagonism expected given the role of terbuthylazine as a driver of this mixture behaviour (see above). It may be that the surfactant(s) selected is not effective in assisting cuticle penetration of the a.s. in *P. oleracea*, but this is highly unlikely given that the species is a major target weed of Winner Top® as immediately indicated in the label^31^, and considering that surfactants are seen as enhancers of biological activity within the formulation thus their selection tends to be careful and well equated^55,58–60^.

## Conclusions

The initial expectations for this study involved the assumption that the a.s. used in Winner Top® would behave as synergists in affecting the selected non-target indicator *L. minor* given the species physiological similarity with target weeds. On the contrary, antagonistic behaviour was found towards the macrophyte. This can be taken as an environmentally protective feature of the combination compared to the single dosing of each a.s. at a first glance, but the recommended application doses clearly deliver edge-of-field surface water PECs above corresponding *L. minor* safety benchmarks. Moreover, antagonistic behaviour was found between the a.s. regarding effects in the major target weed *P. oleracea*, with nicosulfuron apparently being useless for the overall herbicide activity, which was surprising given that a.s. are assumed synergists in the label of the commercial formulation^31^. Still, we were able to signal alternative a.s. formulation options. There were no combination ratios effective against *P. oleracea* that can concomitantly ensure environmental safety. Our results rather show that terbuthylazine alone, dosed at concentrations 10-fold lower than those involved in recommended application doses, is effective in controlling *P. oleracea* and translates into safe PEC levels, i.e. representing lessened environmental hazardous potential.

The huge costs of research and development to place new a.s. in the market - which have been increasing due to a major contribution of environmental chemistry and toxicology regulatory demands^63,64^- have been making the reformulation of existent ones an option since it can be a time- and cost-effective alternative^21^. Still, research and development stages (see Husby *et al*.^65^ for an overview on typical product development stages), namely those involving laboratory and greenhouse tests, eventually field trials, for confirming the biological activity of the renewed product should not be disregarded. This applies also to the formulation solutions applied following the establishment of the a.s. combination. In fact, formulation technology is seen as an enabling arena within agrochemicals development, adding value to the products as to their stability, convenience, human and environmental safety, but primarily by enhancing the a.s. efficacy^56,61^. On the contrary, our results suggest that the formulants used in Winner Top® rather spoil the a.s. efficacy. They indicate further that assessment methods merely based in predictive approaches (e.g. *in silico* tools and QSAR modelling), as those that have been for long used to assist formulation design^66–69^, may be short in capturing the actual efficacy of the designed product and the most favourable formulation for further development, both from an economic (important savings would be made by producing a single-way terbuthylazine formulation) and an environmental (PEC references can apparently be met with formulation changes) point of view. It is worth remarking in this context that our workflow considered simple and affordable (time- and cost-effective) laboratory testing, as well as modelling tools (FOCUS and mixture toxicity assessment tools) of free web access with familiar interfaces.

## Methods

### Chemicals

The chemicals used in the toxicity assays were the commercial formulation Winner Top® (Selectis®, Portugal), terbuthylazine (Pestanal®, Sigma-Aldrich®, Steinheim) and nicosulfuron (Pestanal®, Sigma-Aldrich®, Steinheim). Winner-Top is a two-way herbicide formulating terbuthylazine (250 g/L) and nicosulfuron (16.75 g/L). Stock solutions were prepared immediately before each assay by dissolving the a.s. or diluting the commercial formulation in culture medium or in tap water for testing with *L. minor* and *P. oleracea*, respectively. No solvent carriers were used.

### Test organisms and general test protocols

Cultures of the non-target aquatic macrophyte *L. minor* were maintained in 500-mL Erlenmeyer vessels filled with ca. 200 mL of Steinberg medium^35^, at 20 °C with a photoperiod of 16 h^L^:8 h^D^ (light intensity: ≈2000 Lux). Cultures were renewed once a week. Growth inhibition tests with *L. minor* followed the OECD guideline 221^35^ adapted to 6-well microplate use^70,71^, at 23 °C, under continuous illumination (intensity: ≈1700 Lux), for 7 days. In brief, tests started by inoculating each well with 3 healthy colonies with 3 fronds each, which were then allowed to grow under defined treatment ranges (Table S1). Each chemical treatment was run in triplicate and 6 replicates were used for the control. After 7 days of exposure, fronds were counted and frond yield was calculated through the difference with inoculating frond number.

Seeds of the weed *P. oleracea* (Flora Lusitana Lda., Portugal) were purchased from a local supplier. Vegetative vigour tests with the target weed *P. oleracea* were run by adapting from the OECD guideline 227^72^, using three replicates per treatment (see Table S1 for treatment ranges). Each replicate was set-up by adding 200 g dry-weight LUFA soil (Speyer, Germany) into a plastic pot (95 cm^2^ circular area) holed at the bottom for the placement of a cotton rope allowing bottom watering throughout the test. Although LUFA is a natural soil widely accepted as a standard matrix for soil toxicity tests, three samples of the test soil were briefly characterized (n = 3): pH in H_2_O = 5.32 ± 0.02 SD, conductivity = 309 µS/cm ± 17.8 SD, water content = 6.18% ± 0.08 SD, loss-on-ignition organic matter content = 2.32% ± 0.22 SD, determined according to standard protocols^73^. LUFA was hence found adequate for testing with *P. oleracea*, after pH adjustment using CaCO_3_ (500 mg/Kg dry-weight soil) to reach the plant optimal range^33^. Ten seeds were evenly distributed within each replicated pot with moistened soil and the pots were then placed at 20 ± 3 °C under a 16 h^L^:8 h^D^ photoperiod (light intensity and humidity of 19600 Lux and 50 ± 2%, respectively). Nutrients (Substral®, Scotts Celaflor GmbH) were added to the bottom watering supply vessel once after seedling (14 mL/L).

Only the first 5 seeds emerging were kept in each replicated pot, and the plants were left to grow for 10 days, i.e. until reaching the 4-true leaf stage (Winner Top® application should be performed while the weed plants have 3–5 leaves^31^). The plants were then treated by spraying over the leaves (Turn’n’ spray, Bürkle, with 1.2 ± 0.1 mL spray volume) with the test solutions (see Table S1 for test concentrations). The concentration of test solutions was adjusted so that all replicates within all treatments could receive the same treatment volume, including in the control, where tap water was used instead. Sixteen days following treatment, the assay was finished, the leaves were counted and shoots were harvested for dry weight records after drying until constant weight at 60 °C. These experimental results were directly analysed via one-way ANOVA, followed by the post-hoc Dunnet test as applicable. This allowed addressing the effects of single and combined treatments of nicosulfuron and terbuthylazine in *P. oleracea* vegetative vigour. A significance level of 0.05 was always used.

### Mixture modelling and mixture toxicity analysis

Frond yield records following single exposure of *L. minor* to terbuthylazine and nicosulfuron were fitted to the nonlinear decay model^28^. Significant fitting was always achieved, with model accuracy being assessed through adjusted coefficient of determination (*r*^2^) and residual distribution^74^; the significance of regressions was validated through by the F-test of overall significance^75^. Prediction of the joint action of chemicals was carried out by integrating these experimental data into the reference mixture models of CA and IA assuming no interaction between the components of the mixture. CA is mathematically represented in equation 1, where C*i* represents the individual concentrations of each component present in the mixture with a total effect of x% and EC*xi* are those concentrations of the components that would alone cause the same effect x*i* as observed for the mixture. IA was calculated by multiplying the probability of non-response of each *i*^th^ component of the mixture, following equation 2, where C*i* represents the individual concentrations of each component in the mixture and E(C*i*) is the effect of C*i* when the *i*^th^ component is dosed singly. The effective mixture concentrations are commonly presented using the dimensionless TU scaling allowing a measure of the toxic strength. The sum of the quotients C*i*/EC50*i* was applied for the purpose following the CA principles for *i*^th^ mixture components^29^.

1 $${\sum }_{i=1}^{n}\frac{Ci}{EC{x}_{i}}=1$$

2 $$Rmix=1-{\coprod }_{i=1}^{n}[1-E(Ci)]$$

Based on the expected response curves resulting from the combination of responses to single chemical exposures, a 5 × 5 incomplete factorial (Ray) design (nicosulfuron x terbuthylazine) was used to test mixtures with *L. minor* (Table S1). The concentrations were established ensuring coverage of a wide range of responses, from minimum to high (see details in e.g.^40^). The experimental responses (*L. minor* frond yield) were compared to the reference CA and IA models to assess the most suitable modelling approach. Deviation functions to the reference models adapted from Jonker *et al*.^29^ were fitted to the experimental data to identify if synergistic/antagonistic (S/A) effects, dose-level (DL) or dose-ratio (DR) dependent effects were more suitable to describe the experimental data. These effects can be denoted by the values of two additional model parameters, ‘a’ and ‘b’, as detailed in Jonker *et al*.^29^. Comparisons between different models fitting significantly to the dataset were carried out using the F-test under the assumption that a simpler model (i.e. with less parameters) represents a better fitting solution than a more complex one; the rank from the simpler to the most complex is as follows: Baseline > S/A > DL > DR. The Akaike’s Information Criterion (AIC) was also calculated as a complementary approach to express model fit in a quickly comparable way, with a lower AIC value denoting higher likelihood associated with the model^76^. Mixture toxicity modelling and analysis was run in a customized MS®Excel® spreadsheet (ToxCalcMix, version 1.0, last rev. 20/01/2016; AJA Nogueira, unpublished).

In order to assess the efficacy of eco-friendly formulations between nicosulfuron and terbuthylazine, the target weed *P. oleracea* was tested against mixtures eliciting up to 20% *L. minor* frond inhibition (mixture EC_20_) estimated based on the best fit of mixture models to the experimental data (see above); note that the EC_20_ is a standard protective benchmark^5^. Namely, application doses of nicosulfuron and terbuthylazine were combined, corresponding to concentrations composing *L. minor* mixture EC_1_, EC_5_ and EC_20_; additional single chemical treatments were added to the treatments range to better complete the exposure design by covering more widely single chemical concentration-response curves (see Table S1). Step 1 (Winner Top® is typically applied only once a year and the present study was not based on any specific transport scenario) of the EU-FOCUS platform (http://eusoils.jrc.ec.europa.eu/projects/stepsonetwo) was used to calculate application doses for testing with *P. oleracea* corresponding to *L. minor* EC_*x*_, assuming that these later correspond to surface water PEC values. FOCUS predicts pesticide residue concentrations in edge-of-field waterbodies following given application doses and taking into account pesticide properties constraining their fate through the soils. Since FOCUS runs backwards compared to our needs (the user inserts the application doses and the platform provides PEC values), a previous calibration step was necessary by simulating several application doses for each a.s. and retrieving corresponding PEC values at day 1 (simulation parameters were retrieved from dedicated EU reports and are exposed in Table S2^36,37^). Obtained regressions were as follows for nicosulfuron (equation 3; n = 9 with *r*^2^ = 1.00) and terbuthylazine (equation 4; n = 7 with *r*^2^ = 1.00).

3 $${\rm{PEC}}\,({{\rm{EC}}}_{x}L.\,minor)=0.3277\times {\rm{application}}\,{\rm{dose}}\,{\rm{targeted}}\,{\rm{at}}\,P.\,oleracea$$

4 $${\rm{PEC}}\,({{\rm{EC}}}_{x}L.\,minor)=0.2747\times {\rm{application}}\,{\rm{dose}}\,{\rm{targeted}}\,{\rm{at}}\,P.\,oleracea$$

The experimental responses (*P. oleracea* dry weight) were compared with CA and IA and then added the deviation functions as described above for *L. minor* mixture toxicity results.

Winner Top® was also tested against *P. oleracea* as an additional non-customized mixture treatment for comparative purposes. The recommended application dose for susceptible species (41.88 g/ha nicosulfuron × 625 g/ha terbuthylazine, corresponding to 2.5 L/ha^31^) was down-ranged allowing direct comparison with one of the combinations between a.s. (marked in Table S1). This comparison was set to gain a complementary insight on the actual role of formulants other than the a.s. in the efficacy of the PPP against one its major target weeds.

### Data availability

All data generated or analysed during this study are generally included in this published article (and its Supplementary Information files). Specific data generated during and/or analysed during the current study are available from the corresponding author on reasonable request.

## Electronic supplementary material

Supplementary information
