# Supplementary material for: Mixture toxicity assisting the design of eco-friendlier plant protection products: a case-study using a commercial herbicide combining nicosulfuron and terbuthylazine
Source: Sci Rep. 2018 Apr 3;8:5547. doi: 10.1038/s41598-018-23883-5 (PMC5883046; doi:10.1038/s41598-018-23883-5)
Supplement: Supplementary file 1 — Supplementary information [file 41598_2018_23883_MOESM1_ESM.pdf]

SUPPLEMENTARY INFORMATION

Mixture toxicity assisting the design of eco-friendlier plant protection products: a case-study using a commercial herbicide combining nicosulfuron and terbuthylazine

By Libânia Queirós, Tânia Vidal, António JA Nogueira, Fernando JM Gonçalves, Joana Luísa Pereira (email: jpereira@ua.pt)

Table S1. Concentrations of nicosulfuron (Nic.) and terbuthylazine (Terb.) used in the toxicity assays within the present study. The left side of the table contains the treatments used in single and mixture exposures of the non-target macrophyte *Lemna minor*, while the right side of the table discloses the treatments used in mixture exposures of the target weed *Portulaca oleracea*. Mixture concentrations are given in μg/L or g/ha for *L. minor* and *P. oleracea*, respectively, and using the dimensionless Toxic Units (TU) scaling. PEC values (corresponding to nicosulfuron and terbuthylazine EC_1, EC_5 and EC_{20} found following single toxicity testing with *L. minor*) that translated into the application doses used in *P. oleracea* toxicity testing were given in addition for clarity. The mixture of active substances allowing direct efficacy comparison with the commercial formulation Winner Top® in tests with *P. oleracea* was marked bold.

Lemna minor							***Portulaca oleracea***						
**Single**		**Mixture exposures**					**Mixture exposures**						
**Nic.**	**Terb.**	**Nic.**		**Terb.**		**Σ TU**	**Nic.**			**Terb.**			**Σ TU**
μg/L	μg/L	μg/L	TU	μg/L	TU		PEC (μg/L)	g/ha	TU	PEC (μg/L)	g/ha	TU	
0	0	0.00	0.00	0.00	0.00	0.00	0.00	0.00	0.00	0.00	0.00	0.00	0.00
1	20	0.00	0.00	36.5	0.50	0.50	0.00	0.00	0.00	(EC_1) 0.95	3.46	0.06	0.06
2	33	0.00	0.00	55.9	0.77	0.77	0.00	0.00	0.00	(EC_5) 4.79	17.5	0.30	0.30
3	52	0.00	0.00	85.7	1.17	1.17	0.00	0.00	0.00	8.24	30.0	0.51	0.51
4	84	0.00	0.00	131	1.80	1.80	0.00	0.00	0.00	10.99	40.0	0.69	0.69
7	134	1.45	0.50	0.00	0.00	0.50	0.00	0.00	0.00	13.74	50.0	0.86	0.86
10	215	1.67	0.58	0.00	0.00	0.58	0.00	0.00	0.00	(EC_{20}) 19.98	72.7	1.25	1.25
17	344	3.40	1.17	0.00	0.00	1.17	(EC_1) 0.29	0.89	0.02	0.00	0.00	0.00	0.02
27	550	5.21	1.80	0.00	0.00	1.80	(EC_1) 0.29	0.89	0.02	(EC_1) 0.95	3.46	0.06	0.08
43		1.45	0.50	36.5	0.50	1.00	(EC_1) 0.29	0.89	0.02	(EC_5) 4.79	17.5	0.30	0.32
69		1.45	0.50	55.9	0.77	1.27	(EC_5) 0.72	2.20	0.05	0.00	0.00	0.00	0.05
		1.45	0.50	85.7	1.17	1.67	(EC_5) 0.72	2.20	0.05	(EC_1) 0.95	3.46	0.06	0.11
		1.67	0.58	36.5	0.50	1.08	(EC_5) 0.72	2.20	0.05	(EC_5) 4.79	17.45	0.30	0.35
		1.67	0.58	55.9	0.77	1.35	(EC_{20}) 1.59	4.86	0.12	0.00	0.00	0.00	0.12
		3.40	1.17	36.5	0.50	1.67	**(EC_{20}) 1.59**	**4.86**	**0.12**	**(EC_{20}) 19.98**	**72.74**	**1.25**	**1.37**
		3.40	1.17	85.7	1.17	2.34	2.46	7.50	0.18	0.00	0.00	0.00	0.18
		5.21	1.80	131	1.80	3.60	4.92	15.0	0.36	0.00	0.00	0.00	0.36
							9.83	30.0	0.71	0.00	0.00	0.00	0.71
							19.66	60.0	1.42	0.00	0.00	0.00	1.42
							39.32	120	2.84	0.00	0.00	0.00	2.84

Table S2. Values used to complete the parametrization required at step 1 of the FOCUS platform for estimating PEC values in surface water based on pesticide application doses. All values but application doses were retrieved from EFSA (2011) for terbuthylazine and EFSA (2007) for nicosulfuron.

	Nicosulfuron	Terbuthylazine
**Water solubility (g/L)**	9.5 (pH 6.7, at 19.7 °C)	8.5 (at 20 ºC)
**K_{OC} /K_{foc} (L/kg)**	21	151
**DT_{50} in soil (days)**	16	19.4
**DT_{50} in water/sediment system (days)**	42.3	69.9
**DT_{50} in water (days)**	65	1000
**DT_{50} in sediment (days)**	14	69.9
**Number of applications per season**	1	1
**Crop type**	Maize	Maize
**Region and season of application**	South Europe, Mar-May	South Europe, Mar-May
**Application doses (g/ha)**	5, 10, 15, 20, 25, 42, 50, 75, 100	50, 100, 250, 325, 500, 625, 750
